# 5-{(2*S*,3*R*,4*S*,5*S*,6*R*)-3,4-Dihydr­oxy-6-hydroxy­meth­yl-3-[(2*S*,3*R*,4*R*,5*R*,6*S*)-3,4,5-trihydr­oxy-6-methyl­tetra­hydro­pyran-2-yloxy]tetra­hydro­pyran-2-yloxy}­-7-hydr­oxy-2-(4-hydroxy­phen­yl)chromen-4-one monohydrate

**DOI:** 10.1107/S1600536808027839

**Published:** 2008-09-06

**Authors:** Yuzhen Chen, Airong Wang, Haiyan Gao, Shengyang Niu

**Affiliations:** a Department of Mathematics, Henan Institute of Science and Technology, Xinxiang 453003, People’s Republic of China; b School of Chemistry and Chemical Engineering, Henan Institute of Science and Technology, Xinxiang 453003, People’s Republic of China; c School of Food Science, Henan Institute of Science and Technology, Xinxiang 453003, People’s Republic of China

## Abstract

In the title compound, C_27_H_30_O_14_·H_2_O, the hydroxy­phenyl ring makes a dihedral angle of 20.05 (11)° with the chromenone ring system. The crystal structure is stabilized by intra- and inter­molecular O—H⋯O hydrogen bonds. The absolute configuration was assigned on the basis of an analagous structure.

## Related literature

For related literature, see: Li *et al.* (2007 ▶).
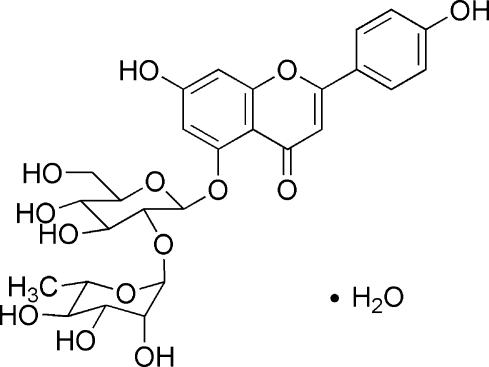


## Experimental

### 

#### Crystal data

C_27_H_30_O_14_·H_2_O
*M* *_r_* = 596.53
Orthorhombic, 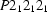

*a* = 7.2392 (14) Å
*b* = 9.832 (2) Å
*c* = 36.782 (7) Å
*V* = 2618.1 (9) Å^3^
*Z* = 4
Mo *K*α radiation
μ = 0.13 mm^−1^
*T* = 113 (2) K
0.08 × 0.06 × 0.04 mm

#### Data collection

Rigaku Saturn CCD area-detector diffractometer
Absorption correction: multi-scan (*CrystalClear*; Rigaku, 2005 ▶) *T*_min_ = 0.990, *T*_max_ = 0.995
20457 measured reflections
3332 independent reflections
2875 reflections with *I* > 2σ(*I*)
*R*_int_ = 0.090

#### Refinement

*R*[*F* ^2^ > 2σ(*F* ^2^)] = 0.045
*wR*(*F* ^2^) = 0.110
*S* = 1.05
3332 reflections
410 parameters
10 restraints
H atoms treated by a mixture of independent and constrained refinement
Δρ_max_ = 0.31 e Å^−3^
Δρ_min_ = −0.29 e Å^−3^

### 

Data collection: *CrystalClear* (Rigaku, 2005 ▶); cell refinement: *CrystalClear*; data reduction: *CrystalClear*; program(s) used to solve structure: *SHELXS97* (Sheldrick, 2008 ▶); program(s) used to refine structure: *SHELXL97* (Sheldrick, 2008 ▶); molecular graphics: *SHELXTL* (Sheldrick, 2008 ▶); software used to prepare material for publication: *CrystalStructure* (Rigaku, 2005 ▶).

## Supplementary Material

Crystal structure: contains datablocks global, I. DOI: 10.1107/S1600536808027839/bt2776sup1.cif

Structure factors: contains datablocks I. DOI: 10.1107/S1600536808027839/bt2776Isup2.hkl

Additional supplementary materials:  crystallographic information; 3D view; checkCIF report

## Figures and Tables

**Table 1:** Hydrogen-bond geometry (Å, °)

*D*—H⋯*A*	*D*—H	H⋯*A*	*D*⋯*A*	*D*—H⋯*A*
O3—H3⋯O15	0.89 (3)	1.80 (3)	2.684 (3)	177 (5)
O6—H6⋯O10^i^	0.82 (3)	2.08 (3)	2.881 (3)	166 (3)
O7—H7⋯O11^i^	0.85 (3)	1.99 (3)	2.817 (3)	164 (4)
O8—H8⋯O14^ii^	0.82 (4)	2.17 (3)	2.821 (3)	136 (3)
O11—H11⋯O2^iii^	0.87 (3)	2.19 (3)	3.006 (3)	158 (3)
O13—H13⋯O2	0.87 (3)	1.79 (3)	2.653 (3)	172 (3)
O14—H14⋯O13^iv^	0.87 (3)	1.76 (3)	2.617 (3)	170 (3)
O15—H15*A*⋯O8	0.80 (3)	2.06 (4)	2.829 (3)	161 (4)
O15—H15*B*⋯O2^v^	0.84 (3)	2.00 (3)	2.820 (4)	168 (4)

Symmetry codes: (i) 
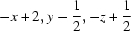
; (ii) 
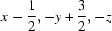
; (iii) 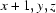; (iv) 
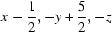
; (v) 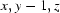.

**Fig. 1.**
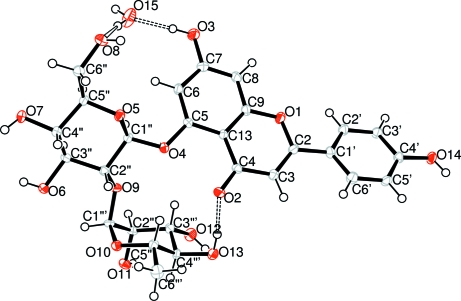
The molecular structure of the title compound with the atom-numbering scheme and 50% probability displacement ellipsoids. The dashed lines indicates H-bonds.

## supplementary crystallographic information

### Comment

The title compound was prepared from compound (II) by hydrolysis (scheme 2).

The hydroxyphenyl ring makes a dihedral angle of 20.05 (11)° with the benzopyranone ring. The two saturated rings adopt chair configurations. The crystal structure is stabilized by intra- and intermolecular O—H···O hydrogen bonds (Table 1), with O···O distances in the range 2.617 (3)–3.006 (3) Å.

### Experimental

Compound (II) was mixed with methanol and K_2_CO_3_. After stirring for 60 min at 40°C, hydrochloric acid was added to make pH 7.0. The mixture was kept for 24 h at 4°C. The resultant white precipitate was separated by filtration, washed with water and dried for X-ray analysis.

### Refinement

In the absence of anomalous scatterers Friedel pairs were merged. The absolute configuration was set according to the literature (Li *et al.*, 2007). The O-bound H atoms were located in a difference map and refined with the restraint O—H = 0.85 (3) Å. All other H atoms were positioned geometrically and refined as riding atoms, with U_iso_(H) = 1.2 U_eq_(CH and CH_2_) and C—H ranging from 0.95–1.0Å or U_iso_(H) = 1.5 U_eq_(CH_3_) and C_methyl_—H = 0.99 Å.

### Crystal data

**Table:**

C_27_H_30_O_14_·H_2_O	*F*(000) = 1256
*M**_r_* = 596.53	*D*_x_ = 1.513 Mg m^−^^3^
Orthorhombic, *P*2_1_2_1_2_1_	Mo *K*α radiation, λ = 0.71073 Å
Hall symbol: P 2ac 2ab	Cell parameters from 5367 reflections
*a* = 7.2392 (14) Å	θ = 2.1–27.1°
*b* = 9.832 (2) Å	µ = 0.13 mm^−^^1^
*c* = 36.782 (7) Å	*T* = 113 K
*V* = 2618.1 (9) Å^3^	Block, white
*Z* = 4	0.08 × 0.06 × 0.04 mm

### Data collection

**Table:**

Rigaku Saturn CCD area-detector diffractometer	3332 independent reflections
Radiation source: rotating anode	2875 reflections with *I* > 2σ(*I*)
confocal	*R*_int_ = 0.090
ω scans	θ_max_ = 27.1°, θ_min_ = 2.1°
Absorption correction: multi-scan (CrystalClear; Rigaku/MSC, 2005)	*h* = −9→9
*T*_min_ = 0.990, *T*_max_ = 0.995	*k* = −12→12
20457 measured reflections	*l* = −30→47

### Refinement

**Table:**

Refinement on *F*^2^	Primary atom site location: structure-invariant direct methods
Least-squares matrix: full	Secondary atom site location: difference Fourier map
*R*[*F*^2^ > 2σ(*F*^2^)] = 0.045	Hydrogen site location: inferred from neighbouring sites
*wR*(*F*^2^) = 0.110	H atoms treated by a mixture of independent and constrained refinement
*S* = 1.06	*w* = 1/[σ^2^(*F*_o_^2^) + (0.0614*P*)^2^ + 0.0488*P*] where *P* = (*F*_o_^2^ + 2*F*_c_^2^)/3
3332 reflections	(Δ/σ)_max_ = 0.001
410 parameters	Δρ_max_ = 0.31 e Å^−^^3^
10 restraints	Δρ_min_ = −0.29 e Å^−^^3^

### Special details

**Table:**

Geometry. All e.s.d.'s (except the e.s.d. in the dihedral angle between two l.s. planes) are estimated using the full covariance matrix. The cell e.s.d.'s are taken into account individually in the estimation of e.s.d.'s in distances, angles and torsion angles; correlations between e.s.d.'s in cell parameters are only used when they are defined by crystal symmetry. An approximate (isotropic) treatment of cell e.s.d.'s is used for estimating e.s.d.'s involving l.s. planes.
Refinement. Refinement of *F*^2^ against ALL reflections. The weighted *R*-factor *wR* and goodness of fit *S* are based on *F*^2^, conventional *R*-factors *R* are based on *F*, with *F* set to zero for negative *F*^2^. The threshold expression of *F*^2^ > σ(*F*^2^) is used only for calculating *R*-factors(gt) *etc*. and is not relevant to the choice of reflections for refinement. *R*-factors based on *F*^2^ are statistically about twice as large as those based on *F*, and *R*- factors based on ALL data will be even larger.

### Fractional atomic coordinates and isotropic or equivalent isotropic displacement parameters (Å^2^)

**Table:**

	*x*	*y*	*z*	*U*_iso_*/*U*_eq_	
O1	0.4843 (3)	0.7580 (2)	0.00818 (5)	0.0211 (5)	
O2	0.5796 (3)	0.9238 (2)	0.10827 (5)	0.0185 (5)	
O3	0.4249 (3)	0.3145 (2)	0.05153 (5)	0.0241 (5)	
H3	0.407 (5)	0.268 (4)	0.0720 (8)	0.036*	
O4	0.5902 (2)	0.6692 (2)	0.13500 (5)	0.0165 (4)	
O5	0.4557 (2)	0.5220 (2)	0.17460 (5)	0.0170 (4)	
O6	0.8762 (3)	0.5651 (2)	0.24894 (5)	0.0205 (4)	
H6	0.909 (5)	0.502 (3)	0.2620 (8)	0.031*	
O7	0.6098 (3)	0.3398 (2)	0.25491 (5)	0.0231 (5)	
H7	0.622 (5)	0.364 (4)	0.2769 (7)	0.035*	
O8	0.2114 (3)	0.2890 (2)	0.17453 (5)	0.0252 (5)	
H8	0.140 (5)	0.342 (4)	0.1646 (9)	0.038*	
O9	0.9173 (2)	0.6586 (2)	0.17330 (5)	0.0167 (4)	
O10	0.9519 (2)	0.8722 (2)	0.20277 (5)	0.0163 (4)	
O11	1.3189 (2)	0.8698 (2)	0.17022 (5)	0.0189 (5)	
H11	1.403 (4)	0.862 (4)	0.1536 (7)	0.028*	
O12	1.1740 (3)	0.8979 (2)	0.09786 (5)	0.0224 (5)	
H12	1.155 (5)	0.981 (3)	0.0934 (9)	0.034*	
O13	0.8811 (3)	1.0723 (2)	0.11859 (5)	0.0219 (5)	
H13	0.779 (4)	1.029 (4)	0.1135 (9)	0.033*	
O14	0.4369 (3)	1.1650 (2)	−0.12150 (5)	0.0212 (5)	
H14	0.406 (5)	1.250 (3)	−0.1214 (9)	0.032*	
O15	0.3844 (4)	0.1713 (3)	0.11315 (6)	0.0375 (6)	
H15A	0.358 (6)	0.199 (5)	0.1329 (8)	0.056*	
H15B	0.438 (6)	0.096 (3)	0.1150 (12)	0.056*	
C2	0.5093 (4)	0.8935 (3)	0.01211 (7)	0.0173 (6)	
C3	0.5473 (4)	0.9489 (3)	0.04519 (7)	0.0190 (6)	
H3A	0.5692	1.0439	0.0469	0.023*	
C4	0.5552 (3)	0.8674 (3)	0.07760 (7)	0.0159 (6)	
C5	0.5377 (3)	0.6235 (3)	0.10095 (7)	0.0149 (6)	
C6	0.4977 (4)	0.4896 (3)	0.09418 (7)	0.0168 (6)	
H6A	0.4939	0.4261	0.1136	0.020*	
C7	0.4621 (4)	0.4463 (3)	0.05842 (7)	0.0186 (6)	
C8	0.4641 (4)	0.5371 (3)	0.02980 (7)	0.0199 (6)	
H8A	0.4449	0.5074	0.0055	0.024*	
C9	0.4948 (4)	0.6725 (3)	0.03761 (7)	0.0178 (6)	
C13	0.5313 (4)	0.7231 (3)	0.07286 (7)	0.0146 (6)	
C1''	0.6285 (4)	0.5673 (3)	0.16087 (7)	0.0150 (5)	
H1''	0.6935	0.4899	0.1488	0.018*	
C2''	0.7487 (3)	0.6234 (3)	0.19134 (7)	0.0154 (6)	
H2''	0.6905	0.7053	0.2027	0.019*	
C3''	0.7742 (4)	0.5101 (3)	0.21932 (7)	0.0167 (6)	
H3''	0.8472	0.4347	0.2080	0.020*	
C4''	0.5868 (4)	0.4542 (3)	0.23160 (7)	0.0167 (6)	
H4''	0.5165	0.5270	0.2446	0.020*	
C5''	0.4779 (4)	0.4074 (3)	0.19843 (7)	0.0158 (6)	
H5''	0.5506	0.3357	0.1855	0.019*	
C6''	0.2879 (4)	0.3529 (3)	0.20634 (7)	0.0203 (6)	
H6''1	0.2948	0.2860	0.2264	0.024*	
H6''2	0.2064	0.4283	0.2142	0.024*	
C1'''	1.0404 (3)	0.7556 (3)	0.18874 (7)	0.0146 (5)	
H1'''	1.1133	0.7111	0.2085	0.018*	
C2'''	1.1695 (3)	0.7886 (3)	0.15686 (7)	0.0159 (6)	
H2'''	1.2187	0.7025	0.1461	0.019*	
C3'''	1.0581 (4)	0.8670 (3)	0.12806 (7)	0.0170 (6)	
H3'''	0.9544	0.8080	0.1195	0.020*	
C4'''	0.9774 (4)	0.9936 (3)	0.14530 (7)	0.0162 (6)	
H4'''	1.0803	1.0497	0.1555	0.019*	
C5'''	0.8485 (3)	0.9518 (3)	0.17625 (7)	0.0163 (6)	
H5'''	0.7459	0.8949	0.1662	0.020*	
C6'''	0.7673 (4)	1.0724 (4)	0.19609 (8)	0.0253 (7)	
H6''3	0.7005	1.0407	0.2177	0.038*	
H6''4	0.6819	1.1207	0.1800	0.038*	
H6''5	0.8668	1.1339	0.2035	0.038*	
C1'	0.4910 (4)	0.9672 (3)	−0.02252 (7)	0.0182 (6)	
C2'	0.5075 (4)	0.8970 (3)	−0.05549 (7)	0.0192 (6)	
H2'	0.5297	0.8018	−0.0555	0.023*	
C3'	0.4916 (4)	0.9662 (3)	−0.08794 (7)	0.0190 (6)	
H3'	0.5052	0.9185	−0.1102	0.023*	
C4'	0.4559 (4)	1.1048 (3)	−0.08840 (7)	0.0179 (6)	
C5'	0.4378 (4)	1.1761 (3)	−0.05556 (7)	0.0202 (6)	
H5'	0.4134	1.2710	−0.0556	0.024*	
C6'	0.4557 (4)	1.1065 (3)	−0.02315 (7)	0.0223 (6)	
H6'	0.4439	1.1545	−0.0009	0.027*	

### Atomic displacement parameters (Å^2^)

**Table:**

	*U*^11^	*U*^22^	*U*^33^	*U*^12^	*U*^13^	*U*^23^
O1	0.0317 (11)	0.0152 (12)	0.0164 (9)	−0.0001 (9)	−0.0024 (8)	0.0025 (9)
O2	0.0233 (10)	0.0176 (13)	0.0144 (9)	−0.0028 (8)	−0.0018 (7)	−0.0026 (8)
O3	0.0339 (12)	0.0184 (14)	0.0201 (10)	−0.0024 (9)	−0.0012 (8)	−0.0014 (9)
O4	0.0217 (9)	0.0151 (12)	0.0126 (8)	−0.0015 (8)	−0.0021 (7)	0.0012 (8)
O5	0.0143 (8)	0.0181 (12)	0.0187 (9)	−0.0011 (8)	−0.0007 (7)	0.0047 (9)
O6	0.0237 (10)	0.0176 (12)	0.0203 (10)	−0.0044 (9)	−0.0101 (8)	0.0030 (9)
O7	0.0326 (11)	0.0202 (12)	0.0164 (9)	−0.0053 (10)	−0.0043 (8)	0.0068 (9)
O8	0.0251 (11)	0.0260 (14)	0.0246 (10)	−0.0073 (9)	−0.0074 (8)	0.0007 (10)
O9	0.0157 (9)	0.0172 (12)	0.0173 (9)	−0.0046 (8)	0.0012 (7)	−0.0033 (8)
O10	0.0179 (9)	0.0153 (12)	0.0157 (8)	0.0015 (8)	−0.0008 (7)	−0.0011 (8)
O11	0.0153 (9)	0.0239 (13)	0.0175 (9)	−0.0033 (8)	0.0003 (7)	−0.0034 (9)
O12	0.0237 (10)	0.0250 (14)	0.0184 (10)	−0.0009 (9)	0.0081 (7)	0.0021 (10)
O13	0.0227 (10)	0.0177 (13)	0.0253 (10)	−0.0020 (9)	−0.0050 (8)	0.0049 (9)
O14	0.0330 (11)	0.0170 (13)	0.0137 (9)	0.0016 (10)	−0.0006 (7)	0.0029 (9)
O15	0.0558 (16)	0.0267 (17)	0.0299 (12)	0.0115 (13)	0.0134 (11)	0.0070 (12)
C2	0.0202 (13)	0.0136 (16)	0.0182 (12)	−0.0012 (11)	0.0004 (9)	0.0007 (12)
C3	0.0242 (13)	0.0166 (17)	0.0162 (12)	−0.0025 (12)	−0.0006 (10)	0.0015 (11)
C4	0.0138 (11)	0.0179 (17)	0.0160 (12)	−0.0024 (11)	−0.0004 (9)	−0.0009 (11)
C5	0.0151 (11)	0.0162 (16)	0.0134 (11)	−0.0001 (11)	−0.0011 (9)	−0.0008 (11)
C6	0.0195 (13)	0.0152 (17)	0.0158 (13)	0.0021 (11)	−0.0011 (9)	0.0017 (12)
C7	0.0182 (12)	0.0164 (17)	0.0213 (13)	−0.0012 (11)	0.0003 (10)	−0.0029 (12)
C8	0.0267 (14)	0.0188 (18)	0.0144 (12)	0.0005 (12)	−0.0027 (10)	−0.0027 (11)
C9	0.0185 (13)	0.0200 (18)	0.0149 (12)	−0.0014 (11)	−0.0014 (9)	0.0024 (12)
C13	0.0171 (12)	0.0136 (16)	0.0132 (12)	−0.0003 (11)	−0.0008 (9)	0.0007 (11)
C1''	0.0172 (12)	0.0134 (15)	0.0143 (11)	−0.0002 (11)	−0.0005 (9)	0.0013 (11)
C2''	0.0155 (12)	0.0155 (17)	0.0153 (12)	−0.0021 (11)	0.0009 (9)	−0.0001 (11)
C3''	0.0189 (13)	0.0154 (17)	0.0160 (12)	−0.0011 (11)	−0.0039 (9)	−0.0017 (11)
C4''	0.0195 (12)	0.0155 (16)	0.0152 (12)	−0.0018 (11)	−0.0010 (9)	0.0017 (11)
C5''	0.0210 (13)	0.0121 (16)	0.0143 (12)	−0.0006 (11)	−0.0006 (9)	0.0025 (11)
C6''	0.0195 (12)	0.0228 (18)	0.0186 (13)	−0.0053 (13)	−0.0021 (9)	0.0022 (13)
C1'''	0.0147 (11)	0.0114 (15)	0.0178 (12)	−0.0007 (11)	−0.0007 (9)	−0.0048 (11)
C2'''	0.0144 (12)	0.0152 (16)	0.0180 (12)	−0.0025 (10)	−0.0013 (9)	−0.0028 (11)
C3'''	0.0166 (12)	0.0195 (17)	0.0150 (12)	−0.0038 (11)	0.0007 (9)	0.0019 (11)
C4'''	0.0177 (12)	0.0156 (17)	0.0152 (12)	−0.0002 (11)	−0.0028 (9)	0.0046 (11)
C5'''	0.0149 (12)	0.0163 (16)	0.0178 (12)	−0.0005 (11)	−0.0001 (9)	0.0009 (12)
C6'''	0.0255 (14)	0.025 (2)	0.0250 (14)	0.0081 (13)	0.0017 (11)	−0.0009 (13)
C1'	0.0202 (13)	0.0202 (18)	0.0142 (12)	0.0015 (12)	−0.0005 (9)	0.0013 (12)
C2'	0.0243 (14)	0.0163 (17)	0.0172 (13)	0.0023 (12)	−0.0004 (10)	−0.0006 (12)
C3'	0.0264 (14)	0.0153 (17)	0.0153 (12)	0.0019 (12)	0.0007 (10)	−0.0023 (11)
C4'	0.0192 (12)	0.0196 (17)	0.0149 (12)	−0.0013 (12)	0.0005 (9)	0.0018 (12)
C5'	0.0306 (15)	0.0114 (17)	0.0186 (13)	0.0010 (12)	0.0000 (11)	−0.0017 (12)
C6'	0.0328 (15)	0.0180 (18)	0.0159 (13)	0.0025 (13)	0.0010 (11)	−0.0031 (12)

### Geometric parameters (Å, °)

**Table:**

O1—C2	1.352 (4)	C8—C9	1.379 (5)
O1—C9	1.373 (3)	C8—H8A	0.9500
O2—C4	1.269 (3)	C9—C13	1.414 (4)
O3—C7	1.347 (4)	C1''—C2''	1.522 (4)
O3—H3	0.89 (2)	C1''—H1''	1.0000
O4—C5	1.384 (3)	C2''—C3''	1.528 (4)
O4—C1''	1.409 (3)	C2''—H2''	1.0000
O5—C1''	1.420 (3)	C3''—C4''	1.532 (4)
O5—C5''	1.436 (3)	C3''—H3''	1.0000
O6—C3''	1.423 (3)	C4''—C5''	1.524 (3)
O6—H6	0.82 (2)	C4''—H4''	1.0000
O7—C4''	1.424 (4)	C5''—C6''	1.505 (4)
O7—H7	0.85 (2)	C5''—H5''	1.0000
O8—C6''	1.439 (3)	C6''—H6''1	0.9900
O8—H8	0.82 (2)	C6''—H6''2	0.9900
O9—C1'''	1.423 (3)	C1'''—C2'''	1.534 (4)
O9—C2''	1.432 (3)	C1'''—H1'''	1.0000
O10—C1'''	1.412 (3)	C2'''—C3'''	1.539 (4)
O10—C5'''	1.457 (3)	C2'''—H2'''	1.0000
O11—C2'''	1.431 (3)	C3'''—C4'''	1.514 (4)
O11—H11	0.86 (2)	C3'''—H3'''	1.0000
O12—C3'''	1.425 (3)	C4'''—C5'''	1.529 (4)
O12—H12	0.85 (3)	C4'''—H4'''	1.0000
O13—C4'''	1.432 (3)	C5'''—C6'''	1.511 (4)
O13—H13	0.87 (2)	C5'''—H5'''	1.0000
O14—C4'	1.360 (3)	C6'''—H6''3	0.9800
O14—H14	0.87 (3)	C6'''—H6''4	0.9800
O15—H15A	0.80 (3)	C6'''—H6''5	0.9800
O15—H15B	0.84 (3)	C1'—C6'	1.393 (5)
C2—C3	1.361 (4)	C1'—C2'	1.400 (4)
C2—C1'	1.472 (4)	C2'—C3'	1.379 (4)
C3—C4	1.437 (4)	C2'—H2'	0.9500
C3—H3A	0.9500	C3'—C4'	1.386 (4)
C4—C13	1.440 (4)	C3'—H3'	0.9500
C5—C6	1.371 (4)	C4'—C5'	1.403 (4)
C5—C13	1.424 (4)	C5'—C6'	1.381 (4)
C6—C7	1.406 (4)	C5'—H5'	0.9500
C6—H6A	0.9500	C6'—H6'	0.9500
C7—C8	1.381 (4)		
			
C2—O1—C9	120.8 (2)	O5—C5''—C4''	108.0 (2)
C7—O3—H3	111 (2)	C6''—C5''—C4''	115.2 (2)
C5—O4—C1''	115.7 (2)	O5—C5''—H5''	108.8
C1''—O5—C5''	111.37 (19)	C6''—C5''—H5''	108.8
C3''—O6—H6	108 (3)	C4''—C5''—H5''	108.8
C4''—O7—H7	112 (3)	O8—C6''—C5''	110.5 (2)
C6''—O8—H8	109 (3)	O8—C6''—H6''1	109.6
C1'''—O9—C2''	120.72 (19)	C5''—C6''—H6''1	109.6
C1'''—O10—C5'''	115.13 (19)	O8—C6''—H6''2	109.6
C2'''—O11—H11	104 (2)	C5''—C6''—H6''2	109.6
C3'''—O12—H12	105 (3)	H6''1—C6''—H6''2	108.1
C4'''—O13—H13	107 (2)	O10—C1'''—O9	113.9 (2)
C4'—O14—H14	116 (2)	O10—C1'''—C2'''	112.6 (2)
H15A—O15—H15B	110 (4)	O9—C1'''—C2'''	102.6 (2)
O1—C2—C3	121.1 (3)	O10—C1'''—H1'''	109.2
O1—C2—C1'	112.4 (2)	O9—C1'''—H1'''	109.2
C3—C2—C1'	126.5 (3)	C2'''—C1'''—H1'''	109.2
C2—C3—C4	121.8 (3)	O11—C2'''—C1'''	108.4 (2)
C2—C3—H3A	119.1	O11—C2'''—C3'''	110.7 (2)
C4—C3—H3A	119.1	C1'''—C2'''—C3'''	108.2 (2)
O2—C4—C3	120.0 (3)	O11—C2'''—H2'''	109.8
O2—C4—C13	123.7 (2)	C1'''—C2'''—H2'''	109.8
C3—C4—C13	116.4 (2)	C3'''—C2'''—H2'''	109.8
C6—C5—O4	122.3 (2)	O12—C3'''—C4'''	112.2 (2)
C6—C5—C13	121.4 (2)	O12—C3'''—C2'''	109.6 (2)
O4—C5—C13	116.3 (2)	C4'''—C3'''—C2'''	109.0 (2)
C5—C6—C7	120.0 (3)	O12—C3'''—H3'''	108.7
C5—C6—H6A	120.0	C4'''—C3'''—H3'''	108.7
C7—C6—H6A	120.0	C2'''—C3'''—H3'''	108.7
O3—C7—C8	118.7 (2)	O13—C4'''—C3'''	110.2 (2)
O3—C7—C6	120.3 (3)	O13—C4'''—C5'''	111.0 (2)
C8—C7—C6	121.0 (3)	C3'''—C4'''—C5'''	109.0 (2)
C9—C8—C7	117.8 (3)	O13—C4'''—H4'''	108.9
C9—C8—H8A	121.1	C3'''—C4'''—H4'''	108.9
C7—C8—H8A	121.1	C5'''—C4'''—H4'''	108.9
O1—C9—C8	114.7 (2)	O10—C5'''—C6'''	107.3 (2)
O1—C9—C13	121.2 (3)	O10—C5'''—C4'''	109.2 (2)
C8—C9—C13	124.1 (3)	C6'''—C5'''—C4'''	112.7 (3)
C9—C13—C5	115.4 (3)	O10—C5'''—H5'''	109.2
C9—C13—C4	118.7 (2)	C6'''—C5'''—H5'''	109.2
C5—C13—C4	125.8 (2)	C4'''—C5'''—H5'''	109.2
O4—C1''—O5	106.9 (2)	C5'''—C6'''—H6''3	109.5
O4—C1''—C2''	110.6 (2)	C5'''—C6'''—H6''4	109.5
O5—C1''—C2''	110.8 (2)	H6''3—C6'''—H6''4	109.5
O4—C1''—H1''	109.5	C5'''—C6'''—H6''5	109.5
O5—C1''—H1''	109.5	H6''3—C6'''—H6''5	109.5
C2''—C1''—H1''	109.5	H6''4—C6'''—H6''5	109.5
O9—C2''—C1''	103.53 (19)	C6'—C1'—C2'	119.1 (3)
O9—C2''—C3''	112.7 (2)	C6'—C1'—C2	121.0 (2)
C1''—C2''—C3''	107.5 (2)	C2'—C1'—C2	119.9 (3)
O9—C2''—H2''	110.9	C3'—C2'—C1'	119.9 (3)
C1''—C2''—H2''	110.9	C3'—C2'—H2'	120.0
C3''—C2''—H2''	110.9	C1'—C2'—H2'	120.0
O6—C3''—C2''	107.5 (2)	C2'—C3'—C4'	120.7 (3)
O6—C3''—C4''	111.7 (2)	C2'—C3'—H3'	119.6
C2''—C3''—C4''	110.7 (2)	C4'—C3'—H3'	119.6
O6—C3''—H3''	109.0	O14—C4'—C3'	117.2 (2)
C2''—C3''—H3''	109.0	O14—C4'—C5'	122.9 (3)
C4''—C3''—H3''	109.0	C3'—C4'—C5'	119.9 (3)
O7—C4''—C5''	107.7 (2)	C6'—C5'—C4'	119.2 (3)
O7—C4''—C3''	110.9 (2)	C6'—C5'—H5'	120.4
C5''—C4''—C3''	109.3 (2)	C4'—C5'—H5'	120.4
O7—C4''—H4''	109.6	C5'—C6'—C1'	121.2 (3)
C5''—C4''—H4''	109.6	C5'—C6'—H6'	119.4
C3''—C4''—H4''	109.6	C1'—C6'—H6'	119.4
O5—C5''—C6''	107.1 (2)		
			
C9—O1—C2—C3	0.7 (4)	O6—C3''—C4''—C5''	175.5 (2)
C9—O1—C2—C1'	−179.5 (2)	C2''—C3''—C4''—C5''	55.7 (3)
O1—C2—C3—C4	−2.6 (4)	C1''—O5—C5''—C6''	−170.6 (2)
C1'—C2—C3—C4	177.6 (2)	C1''—O5—C5''—C4''	64.7 (3)
C2—C3—C4—O2	−175.6 (3)	O7—C4''—C5''—O5	−179.4 (2)
C2—C3—C4—C13	3.5 (4)	C3''—C4''—C5''—O5	−58.9 (3)
C1''—O4—C5—C6	9.7 (3)	O7—C4''—C5''—C6''	60.8 (3)
C1''—O4—C5—C13	−169.0 (2)	C3''—C4''—C5''—C6''	−178.6 (3)
O4—C5—C6—C7	−174.0 (2)	O5—C5''—C6''—O8	70.4 (3)
C13—C5—C6—C7	4.6 (4)	C4''—C5''—C6''—O8	−169.4 (2)
C5—C6—C7—O3	179.2 (3)	C5'''—O10—C1'''—O9	−61.0 (3)
C5—C6—C7—C8	−1.0 (4)	C5'''—O10—C1'''—C2'''	55.4 (3)
O3—C7—C8—C9	177.3 (3)	C2''—O9—C1'''—O10	−43.8 (3)
C6—C7—C8—C9	−2.5 (4)	C2''—O9—C1'''—C2'''	−165.8 (2)
C2—O1—C9—C8	179.2 (2)	O10—C1'''—C2'''—O11	66.0 (3)
C2—O1—C9—C13	0.2 (4)	O9—C1'''—C2'''—O11	−171.1 (2)
C7—C8—C9—O1	−176.4 (2)	O10—C1'''—C2'''—C3'''	−54.2 (3)
C7—C8—C9—C13	2.5 (4)	O9—C1'''—C2'''—C3'''	68.7 (3)
O1—C9—C13—C5	179.7 (2)	O11—C2'''—C3'''—O12	62.0 (3)
C8—C9—C13—C5	0.9 (4)	C1'''—C2'''—C3'''—O12	−179.3 (2)
O1—C9—C13—C4	0.8 (4)	O11—C2'''—C3'''—C4'''	−61.2 (3)
C8—C9—C13—C4	−178.1 (3)	C1'''—C2'''—C3'''—C4'''	57.5 (3)
C6—C5—C13—C9	−4.5 (4)	O12—C3'''—C4'''—O13	55.4 (3)
O4—C5—C13—C9	174.2 (2)	C2'''—C3'''—C4'''—O13	176.9 (2)
C6—C5—C13—C4	174.4 (3)	O12—C3'''—C4'''—C5'''	177.5 (2)
O4—C5—C13—C4	−6.9 (4)	C2'''—C3'''—C4'''—C5'''	−61.0 (3)
O2—C4—C13—C9	176.5 (2)	C1'''—O10—C5'''—C6'''	−179.1 (2)
C3—C4—C13—C9	−2.5 (4)	C1'''—O10—C5'''—C4'''	−56.6 (3)
O2—C4—C13—C5	−2.3 (4)	O13—C4'''—C5'''—O10	−179.9 (2)
C3—C4—C13—C5	178.7 (2)	C3'''—C4'''—C5'''—O10	58.5 (3)
C5—O4—C1''—O5	−80.2 (3)	O13—C4'''—C5'''—C6'''	−60.8 (3)
C5—O4—C1''—C2''	159.1 (2)	C3'''—C4'''—C5'''—C6'''	177.7 (2)
C5''—O5—C1''—O4	173.8 (2)	O1—C2—C1'—C6'	160.3 (3)
C5''—O5—C1''—C2''	−65.7 (3)	C3—C2—C1'—C6'	−19.9 (4)
C1'''—O9—C2''—C1''	158.0 (2)	O1—C2—C1'—C2'	−19.2 (3)
C1'''—O9—C2''—C3''	−86.2 (3)	C3—C2—C1'—C2'	160.6 (3)
O4—C1''—C2''—O9	−64.0 (3)	C6'—C1'—C2'—C3'	0.9 (4)
O5—C1''—C2''—O9	177.7 (2)	C2—C1'—C2'—C3'	−179.6 (3)
O4—C1''—C2''—C3''	176.6 (2)	C1'—C2'—C3'—C4'	−1.1 (4)
O5—C1''—C2''—C3''	58.3 (3)	C2'—C3'—C4'—O14	−178.2 (2)
O9—C2''—C3''—O6	70.4 (3)	C2'—C3'—C4'—C5'	0.7 (4)
C1''—C2''—C3''—O6	−176.2 (2)	O14—C4'—C5'—C6'	178.8 (3)
O9—C2''—C3''—C4''	−167.4 (2)	C3'—C4'—C5'—C6'	0.0 (4)
C1''—C2''—C3''—C4''	−53.9 (3)	C4'—C5'—C6'—C1'	−0.2 (4)
O6—C3''—C4''—O7	−66.0 (3)	C2'—C1'—C6'—C5'	−0.3 (4)
C2''—C3''—C4''—O7	174.2 (2)	C2—C1'—C6'—C5'	−179.7 (3)

### Hydrogen-bond geometry (Å, °)

**Table:**

*D*—H···*A*	*D*—H	H···*A*	*D*···*A*	*D*—H···*A*
O3—H3···O15	0.89 (3)	1.80 (3)	2.684 (3)	177 (5)
O6—H6···O10^i^	0.82 (3)	2.08 (3)	2.881 (3)	166 (3)
O7—H7···O11^i^	0.85 (3)	1.99 (3)	2.817 (3)	164 (4)
O8—H8···O14^ii^	0.82 (4)	2.17 (3)	2.821 (3)	136 (3)
O11—H11···O2^iii^	0.87 (3)	2.19 (3)	3.006 (3)	158 (3)
O13—H13···O2	0.87 (3)	1.79 (3)	2.653 (3)	172 (3)
O14—H14···O13^iv^	0.87 (3)	1.76 (3)	2.617 (3)	170 (3)
O15—H15A···O8	0.80 (3)	2.06 (4)	2.829 (3)	161 (4)
O15—H15B···O2^v^	0.84 (3)	2.00 (3)	2.820 (4)	168 (4)

Symmetry codes: (i) −*x*+2, *y*−1/2, −*z*+1/2; (ii) *x*−1/2, −*y*+3/2, −*z*; (iii) *x*+1, *y*, *z*; (iv) *x*−1/2, −*y*+5/2, −*z*; (v) *x*, *y*−1, *z*.

## References

[bb1] Li, W., Deng, Y. L., Dai, R. J., Yu, Y. H., Saeed, M. K., Li, L., Meng, W. W. & Zhang, X. S. (2007). *J. Pharm. Biomed. Anal.* **45**, 38–46. 10.1016/j.jpba.2007.05.027 17651936

[bb2] Rigaku (2005). *CrystalClear* and *CrystalStructure* Rigaku Corporation, Tokyo, Japan.

[bb3] Sheldrick, G. M. (2008). *Acta Cryst.* A**64**, 112–122. 10.1107/S0108767307043930 18156677

